# Enhanced isolation of influenza viruses in qualified cells improves the probability of well-matched vaccines

**DOI:** 10.1038/s41541-021-00415-3

**Published:** 2021-12-09

**Authors:** Heidi Peck, Karen L. Laurie, Steve Rockman, Vivian Leung, Hilda Lau, Sally Soppe, Cleve Rynehart, Chantal Baas, Heidi Trusheim, Ian G. Barr

**Affiliations:** 1 WHO Collaborating Centre for Reference and Research on Influenza, VIDRL, The Peter Doherty Institute for Infection and Immunity, Parkville, VIC Australia; 2 Seqirus Ltd, Parkville, VIC Australia; 3 Department of Immunology and Microbiology, The University of Melbourne, Parkville, VIC Australia; 4 IDT Biologika GmbH, Dessau, Germany

**Keywords:** Cell vaccines, Influenza virus

## Abstract

Influenza vaccines are utilised to combat seasonal and pandemic influenza. The key to influenza vaccination currently is the availability of candidate vaccine viruses (CVVs). Ideally, CVVs reflect the antigenic characteristics of the circulating virus, which may vary depending upon the isolation method. For traditional inactivated egg-based vaccines, CVVs are isolated in embryonated chicken eggs, while for cell-culture production, CVV’s are isolated in either embryonated eggs or qualified cell lines. We compared isolation rates, growth characteristics, genetic stability and antigenicity of cell and egg CVV’s derived from the same influenza-positive human clinical respiratory samples collected from 2008–2020. Influenza virus isolation rates in MDCK33016PF cells were twice that of eggs and mutations in the HA protein were common in egg CVVs but rare in cell CVVs. These results indicate that fully cell-based influenza vaccines will improve the choice, match and potentially the effectiveness, of seasonal influenza vaccines compared to egg-based vaccines.

## Introduction

Influenza is a highly contagious, febrile respiratory illness that is responsible for an estimated 300,000–650,000 deaths annually^1,2^. Vaccination is the most effective treatment to prevent infection. For the majority of influenza vaccines that are currently produced, the supply of suitable influenza seed viruses for vaccine production depends on the isolation and propagation of influenza viruses from original clinical respiratory samples (OCS).

Traditionally, influenza vaccines have been produced by propagating the viruses required in embryonated chicken eggs. Clinical samples are directly inoculated into the amniotic or allantoic cavities of eggs^3^. Further manipulations on these initial virus isolates, such as by reassorting with laboratory-adapted viruses, are then required to generate high yielding viruses suitable for vaccine production^4^. Alternatively, the isolation and propagation of influenza viruses can be performed in continuous cell culture that has been qualified for use in human vaccines^5^. Concurrent to virus isolation for vaccine candidate production, influenza viruses are typically propagated in cell lines in surveillance laboratories to isolate viruses for antigenic analysis. Many continuous cell lines have been shown to support the growth of influenza viruses, such as Madin–Darby Canine Kidney (MDCK)^6–8^, MDCK-SIAT1 (MDCK cells that overexpress α-2,6-sialoglycans)^9^, MDCK-SIAT1-TMPRSS2 (MDCK-SIAT1 cells expressing transmembrane protease, serine 2)^10^, hCK (MDCK cells that overexpress α-2,6-sialoglycans and have extremely low expression of α-2,3-sialoglycans)^11^, African green monkey kidney (Vero)^12,13^, baby hamster kidney (BHK-21)^6^, SJPL (St Jude porcine lung cells)^14^, Lewis lung carcinoma monkey kidney (LLC-MK2)^7^, swine nasal epithelial cells (siNEC) and tracheal epithelial cells (siTEC)^15^ and EB66^[®16^ cells. Among these, MDCK and MDCK-SIAT1 cell lines are commonly used as they are highly permissive to influenza viruses, easy to cultivate and progeny virus contain few modifications from the OCS^9^. Currently, the majority of influenza virus vaccines are still manufactured in embryonated hen’s eggs^17,18^, although alternative non-egg host systems, such as the use of mammalian cells and recombinant protein vaccines have been licensed (reviewed in ref. ^5^).

It is well recognised that the propagation of influenza viruses in eggs can result in the selection of egg-adapted viruses with mutations in the influenza virus surface protein, haemagglutinin (HA)^19–21^. Mutations in the antigenic sites of the HA protein may alter the immune epitopes of the virus and alter the antigenicity of the virus^21–25^. This has the potential to reduce the effectiveness of the influenza vaccine^22,26,27^. In the past eleven years, A(H3N2) viruses have antigenically drifted rapidly resulting in more frequent updates to the recommendation for the A(H3N2) component of the vaccine compared to A(H1N1)pdm09 and influenza B (Victoria or Yamagata)^28^. Egg adaptation of viruses occurs in both influenza A and B viruses, but are most common for A(H3N2) viruses^29^ and are in part responsible for the reduced efficacy of this vaccine component^22,26,27^. Thus, the challenge to isolate A(H3N2) candidate vaccine viruses (CVV’s) in eggs that are antigenically well matched to circulating strains and do not contain undesirable egg adaptations in the HA protein is still ongoing.

The aim of this study was to assess the isolation rates, genetic and antigenic characteristics and growth properties of influenza viruses isolated in a proprietary MDCK cell line, MDCK33016PF^30^, compared to embryonated hens eggs, for potential use as influenza CVV’s. MDCK33016PF cells are highly permissive to influenza viruses whilst having limited ability to propagate other human viruses, a characteristic similar to embryonated hen’s eggs^31–33^. It was hypothesised that propagation of human influenza viruses in mammalian cells would result in improved isolation of viruses with fewer mutations in the HA compared to isolation in embryonated hen’s eggs.

## Results

### Higher isolation rates of human seasonal influenza viruses in MDCK33016PF cells compared to embryonated hen’s eggs

**Fig. 1:**
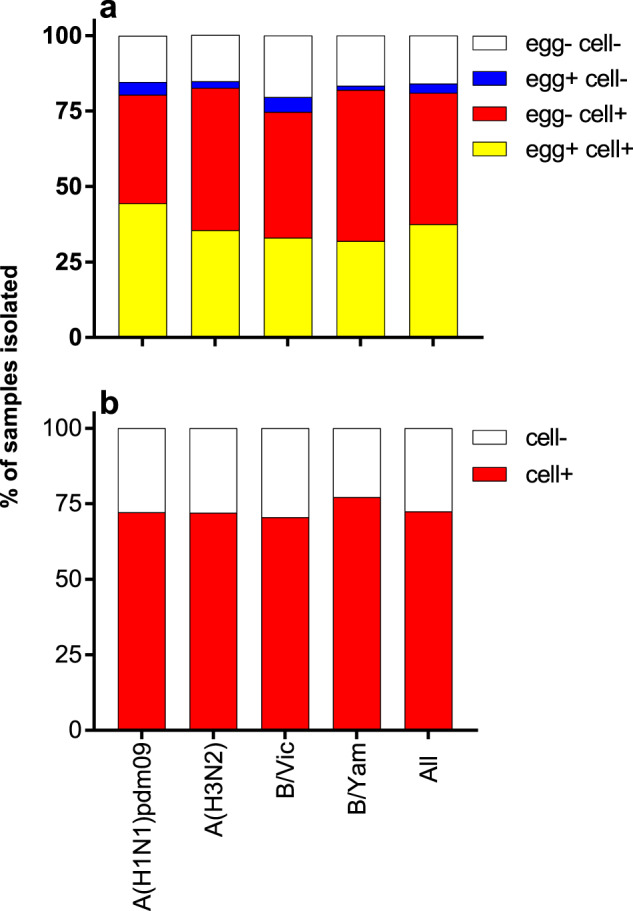
The proportion recovery in each culture type of viruses grouped by subtype/lineage and of all samples combined. **a** 895 samples were inoculated into both cells and eggs and **b** 369 samples were inoculated into cells only.

The isolation rate of human viruses from OCS was directly compared between cells and eggs from 2008–2020. OCS from 895 individuals were inoculated into both substrates and were assessed for virus growth by Haemagglutination (HA) assay. Overall, 336 samples (37.5%) were isolated in both cells and eggs, 389 samples (43.5%) were isolated in cells only, 27 samples were isolated in eggs only (3%) and 143 (16%) samples were not isolated in either substrate (Fig. 1a). Samples were twice as likely to be isolated in cells (81.0%) than eggs (40.6%) (*p* < 0.00001).

For all influenza A subtypes and B virus lineages, a higher proportion of viruses were isolated in cells than in eggs (Fig. 1a). Over 80% of viruses from each subtype/lineage were isolated in cells (range 74.8–82.6%) (Fig. 1a). Almost half of A(H1N1)pdm09 viruses were isolated in eggs (48.7%), whilst lower proportions of A(H3N2) (37.7%), B/Victoria (37.9%) and B/Yamagata (33.3%) viruses were isolated in eggs (Fig. 1a).

During this study, an additional 369 OCS were inoculated into cells only giving an overall isolation rate in cells for all subtypes/lineages of 78.5% (range 73.5–80.4%, Fig. 1b).

**Fig. 2:**
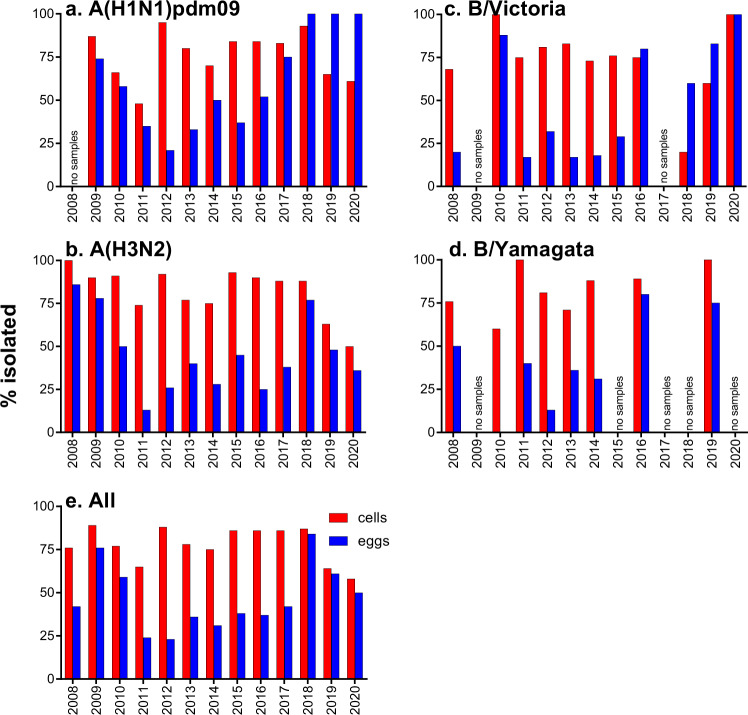
Isolation rates of all human influenza viruses tested in cells and eggs. Proportion recovered in cells (red bar) or eggs (blue bar) by year of sample collection is shown for A(H1N1)pdm09 (**a**), A(H3N2) (**b**), B/Victoria (**c**), B/Yamagata (**d**) and all (**e**) viruses.

Analysis by sample year and subtype/lineage showed that isolation of viruses in cells was more consistent than eggs (Fig. 2). There was significantly less variation in the isolation rate of viruses in cells as compared to eggs, from year to year, for A(H1N1)pdm09 (*p* = 0.0153) and B/Yamagata (*p* = 0.0462) viruses. There was no statistically significant difference in variation in the isolation rate of viruses in cells as compared to eggs, from year to year for A(H3N2) (*p* = 0.0600) and B/Vic viruses (*p* = 0.0961). Early in the study, the isolation rates were comparable between cells and eggs, but very low isolation rates in eggs (less than 50%) were observed between 2011–2013, and in 2015 for A(H1N1)pdm09 viruses, for 2011–2017, 2020 for A(H3N2) and 2011–2015 for B/Victoria and for 2010–2014 for B/Yamagata viruses (Fig. 2, see Supplementary Table 1 for raw data).

In the first few years after the 2009 pandemic, isolation of A(H1N1)pdm09 viruses in embryonated eggs by allantoic inoculation of influenza-positive OCS was quite successful. However, by 2012, A(H1N1)pdm09 isolation rates had fallen to around 20% and the inoculation method for A(H1N1)pdm09 OCS reverted to the methods used prior to 2009 for seasonal A(H1N1) viruses. They consisted of initial isolation in the amniotic cavity followed by passaging of viruses in the allantoic cavity of embryonated hen’s eggs. From 2013, egg isolation rates for A(H1N1)pdm09 containing samples improved and by 2017 had reached 75% or greater with some years having higher isolation rates in eggs than in cells (Fig. 2a), although only a small number of isolations were attempted in eggs in these years (isolations for 2018: eggs- 17/17, cells – 41/44; 2019: eggs – 4/4, cells – 31/48; 2020: eggs – 2/2, cells – 11/18).

**Fig. 3:**
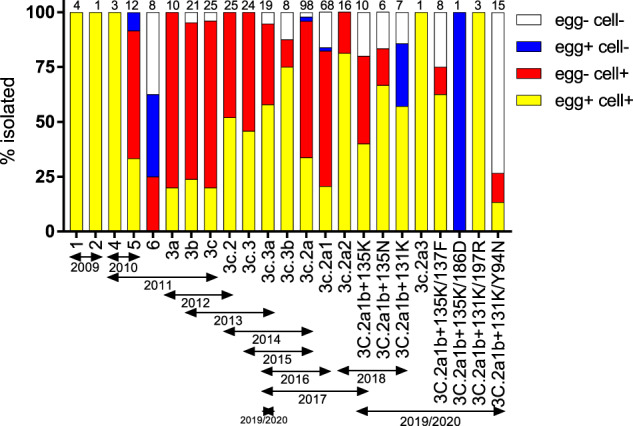
Isolate rates of A(H3N2) viruses tested in cells and eggs by clade. Numbers at the top of the bars indicate the number of viruses attempted in each clade. Virus clades were determined by sequencing the HA gene.

The genetic and antigenic complexity of A(H3N2) has increased in recent years resulting in the designation of multiple genetic clades and sub-clades based on the HA gene^34^. The isolation rate was assessed by clade for A(H3N2) viruses where sequences were available (Fig. 3). At least one virus was isolated in eggs and in cells for each clade, except for 3c2a1b/131K/186D, where only one OCS was received and only grew in eggs. Viruses in the 3a, 3b and 3c clades, which circulated in 2011–2013, were isolated in very high proportions (95–100%) in cells but only at low proportions (20–24%) in eggs. Similarly, two-thirds of viruses in the 3c.2a and 3c.2a1 clades, which circulated in 2014–2017, were isolated only in cells. This is in contrast to viruses early in the study (2009–2010), or more recently (2018–2020), which have been more readily isolated in both cells and eggs.

### Differential binding of turkey and guinea pig red blood cells to virus isolates over time

**Fig. 4:**
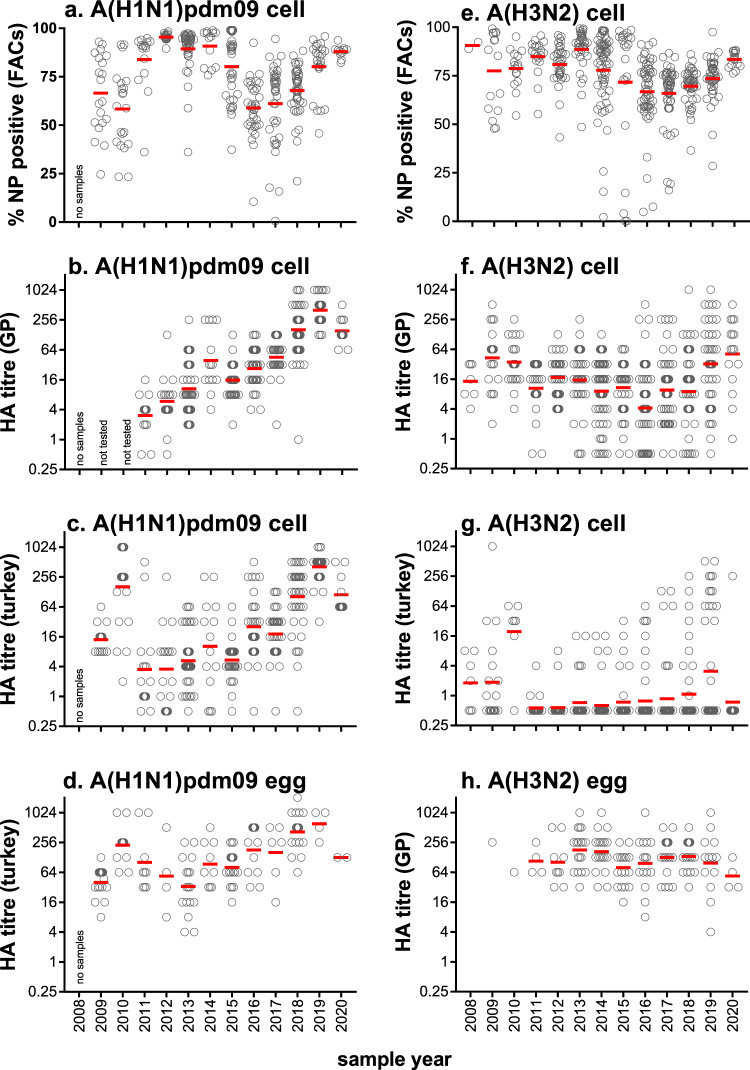
HA titres of influenza A isolates to GP and turkey RBC, from 2008–2020. **a**, **e** Cell-grown A(H1N1)pdm09 and A(H3N2) viruses were assessed for infectivity of cells by flow cytometry. The red bar indicates the mean. HA titres of cell-grown isolates to GP RBC (**b**, **f**) and turkey RBC (**c**, **g**). **d** The HA titre of egg-grown A(H1N1)pdm09 viruses was measured using turkey RBC. **h** The HA titre of egg-grown A(H3N2) viruses was measured using GP RBC. (**b**–**d**, **f**–**h**) Red bar indicates geometric mean titre).

Chicken, turkey and mammalian red blood cells (RBC) have traditionally been used to detect and quantitate influenza viruses for several decades. RBC from different species express different levels of glycan linkages making them useful to assess changes in the HA receptor binding preferences^24^. Guinea pig (GP) RBC express more α2,6-linked sialylated N-glycans than turkey RBC and chicken RBC, which have more α2,3-linked sialylated N-glycans^35^. Throughout this study the titre of cell-grown viruses was measured by HA assay using GP and turkey RBC, effectively assessing the binding preferences of HA over time. Cell-derived A(H1N1)pdm09 viruses are consistently bound to both GP and turkey RBC with titres increasing from 2011 to 2019 following the introduction of the pandemic A(H1N1) virus in 2009 (Fig. 4b, c). Increases in HA titres for A(H1N1)pdm09 viruses grown in eggs was also observed (Fig. 4d) from 2014. The proportion of MDCK33016PF cells infected with a virus, as determined by flow cytometry detecting nucleoprotein (NP) expression as a marker of virus infection, was greater than 50% for all years (Fig. 4a). The majority of A(H3N2) viruses (87%) lost the ability to bind to turkey RBC from 2011–2018 (Fig. 4g) yet retained binding to GP RBC (Fig. 4f). A consistent level of infection in cells was maintained (Fig. 4e) and consistent HA titres were observed in egg-grown viruses as measured with GP RBC (Fig. 4h). These data indicate a change in the binding preference of the H3 HA away from avian-like receptors towards mammalian-like receptors during the study period. HA titres for cell-grown influenza B viruses were consistent over time (see Supplementary Information, Supplementary Fig. 1).

### Comparison of virus growth for cell and egg isolates

**Fig. 5:**
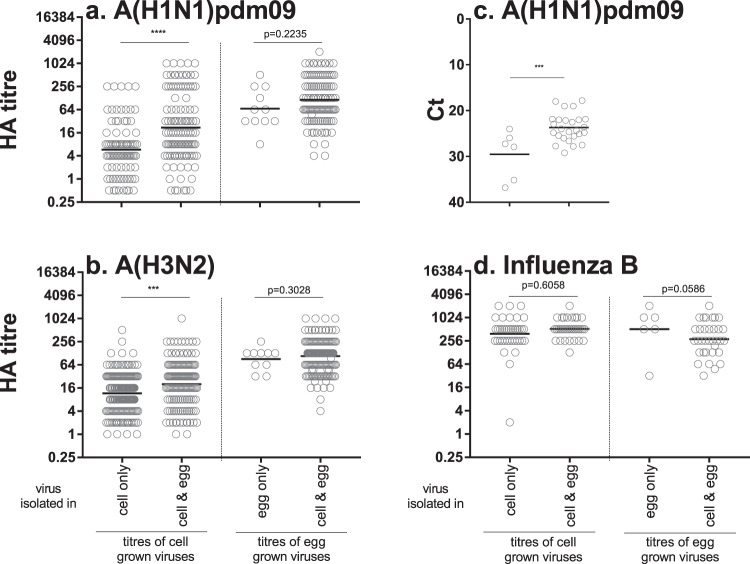
Assessment of HA titre for cell-grown or egg-grown viruses. Viruses were inoculated into both cells and eggs and growth was assessed, as per Fig. 1a. Cell-grown viruses were grouped by viruses that were isolated only in cells as compared to viruses that were isolated in cells and eggs. Egg-grown viruses were grouped by viruses that were isolated only in eggs as compared to viruses that were isolated in cells and eggs. The HA titres were plotted for A(H1N1)pdm09 (**a**) and influenza B (**d**) as detected by turkey RBC and A(H3N2) (**b**) as detected by GP RBC. **c** The Ct of vRNA for a subset of A(H1N1)pdm09 samples was measured. ***p < 0.001, ****p < 0.0001. The difference in HA titres or Ct was assessed using an unpaired *t*-test.

The HA titres of viruses that grew in cells only, eggs only or both cells and eggs were compared. Influenza A viruses that were isolated in both cells and eggs had higher HA titres in cells than influenza A viruses that grew in cells only (Fig. 5a, b). The amount of viral RNA (as determined by real-time PCR cycle threshold values) was also higher in the OCS of A(H1N1)pdm09 samples that were isolated both in cells and eggs as compared to A(H1N1)pdm09 samples isolated in cells alone (Fig. 5c). These data indicate that the OCS’s containing higher amounts of virus were more often isolated in both cells and eggs whilst samples containing less virus were more likely to be isolated only in cells. Influenza A viruses that were isolated in both cells and eggs had similar HA titres in eggs as influenza A viruses that grew in eggs only (Fig. 5a, b). There was no statistical difference in titre for influenza B viruses that were isolated in cells alone compared to those isolated in both cells and eggs (Fig. 5d).

### Comparison of mutations in the HA and NA genes in the cell and egg isolates

**Fig. 6:**
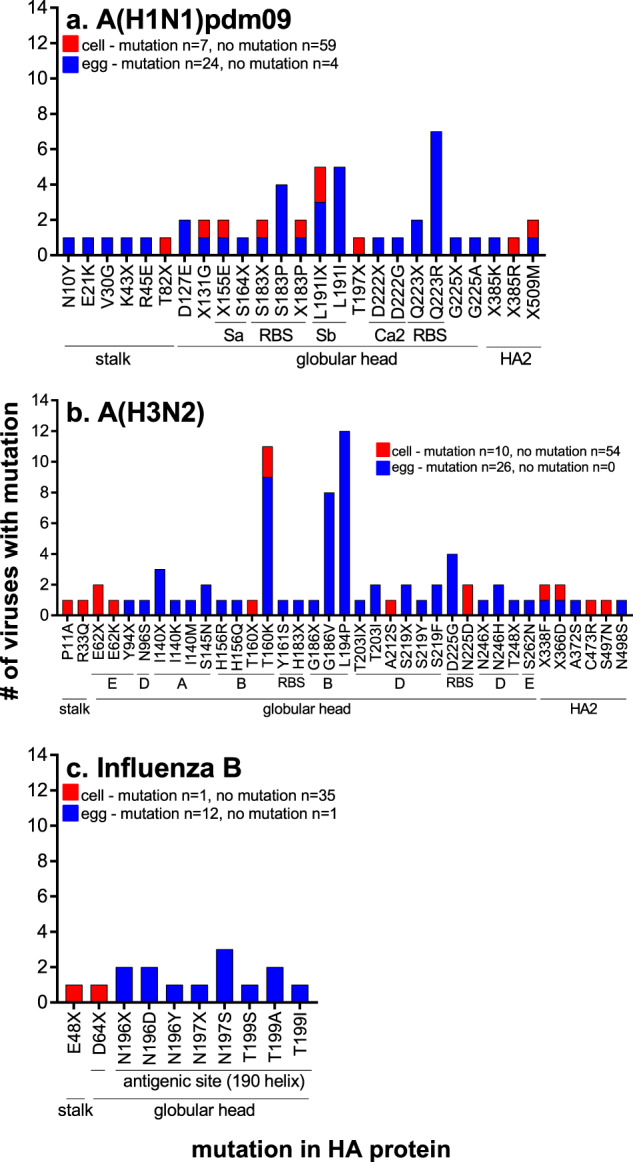
Assessment of mutations in the HA protein following virus isolation. The frequency of mutations in HA protein of viruses isolated in cells and eggs for A(H1N1)pdm09 (**a**), A(H3N2) (**b**), B/Victoria and B/Yamagata (**c**) as compared to the OCS. The total number of cell and egg virus isolates with HA gene sequenced and the number of viruses with and without any mutation in the HA is indicated on each graph. The location of each mutation in the HA protein is indicated below the x-axis, as determined using^26,58–60^.

A subset of viruses (*n* = 68) was assessed for the stability of the HA sequence after isolation and subsequent passage in both eggs and cells by consensus Sanger sequencing of the HA gene. Non-synonymous mutations in the HA gene were observed in 93% (63/68) of viruses passaged in eggs and 10% (7/68) of viruses passaged in cells, as compared to the HA gene sequence of the OCS (Fig. 6). The HA sequences were assessed for a further 98 viruses passaged in cells only, and non-synonymous mutations in the HA were observed in 11% (11/98) of viruses; taken together, mutations were observed in 11% (18/166) of all sequenced viruses passaged in cells (Fig. 6). Of the seven viruses with mutations observed in both cells and eggs, common mutations following cell- and egg-passage were observed in four of the viruses (see Supplementary Information, Supplementary Table 2). Of the four viruses, three had polymorphisms in the HA of the OCS, that when isolated selected the same amino acid in both cells and eggs, indicating that there was no selection by the isolation substrate (see Supplementary Information, Supplementary Table 2). Following passage in eggs, the proportion of viruses with mutations was similar for both influenza A subtypes and influenza B viruses (A(H1N1)pdm09—86% (24/28), A(H3N2)—100% (26/26), B—93% (13/14)). Influenza B viruses were less likely to acquire mutations in the HA following passage in cells (3% 1/36), than influenza A viruses (A(H1N1)pdm09—11% (7/66), A(H3N2)—16% (10/64) (Fig. 6). The NA gene sequences were also assessed by consensus Sanger sequencing. Adaptive mutations in the NA gene of egg-grown viruses was less common, observed in 40% (6/15) of A(H1N1)pdm09 viruses, 8% (2/26) of A(H3N2) viruses and no (0/9) influenza B viruses. Adaptive mutations in the NA gene of cell-grown viruses was rare, observed only in 7% (1/15) of A(H1N1)pdm09 viruses and none in A(H3N2) (0/25) or influenza B (0/9) viruses (Supplementary Information, Supplementary Table 2).

Mutations were located mainly in the head of the HA protein in both egg and cell passaged viruses (Fig. 6 and Supplementary Information, Supplementary Table 2). Over twice as many mutation sites were located in antigenic sites for A(H3N2) viruses passaged in eggs (65% 13/20) than passaged in cells (30% 3/10) (Fig. 6b), and for influenza B viruses (eggs 100% 3/3, cells 0% 0/2)- (Fig. 6c). Similar proportions of mutation sites were located in the antigenic sites of H1 HA for egg (23.5% 4/17) and cell (25% 2/8) passaged viruses (Fig. 6a). One-quarter of mutations observed following passage in cells and in eggs resulted in mixed amino acids in the HA protein (Fig. 6).

Half the amino acid sites that mutated following passage in eggs (19/38) were shared among more than one virus (Fig. 6). Seven mutations were seen in at least four influenza A viruses each and are indicative of common egg-adaptation mutations (S183P, L191I and Q223R in A(H1N1)pdm09 viruses, T160K, G186V, L194Pand S219Y/F in A(H3N2) viruses)^20,22,24,29,36^ (Fig. 6a, b). Mutations observed following passage in cells were only found in one or two viruses (Fig. 6a, b). The N196D/Y/X mutations in B/Yamagata viruses is structurally equivalent to the N197S/X mutation in B/Victoria viruses and was observed in nine egg-passaged influenza B viruses (Fig. 6c).

Overall, these data indicate that cell-grown viruses are more representative of currently circulating strains in humans than egg-grown influenza viruses.

### Egg adaptations in the HA protein affect the antigenicity of egg-passaged viruses

The impact of egg adaptation/s in the HA protein on the antigenicity of A(H3N2) viruses was assessed by the haemagglutination inhibition (HI) assay against a panel of post-influenza vaccination human sera. The reactivity of sera from patients that had received the egg-grown influenza virus vaccine was tested against cell- and egg-grown pairs of viruses from different A(H3N2) genetic clades that circulated from 2014–2017.

**Fig. 7:**
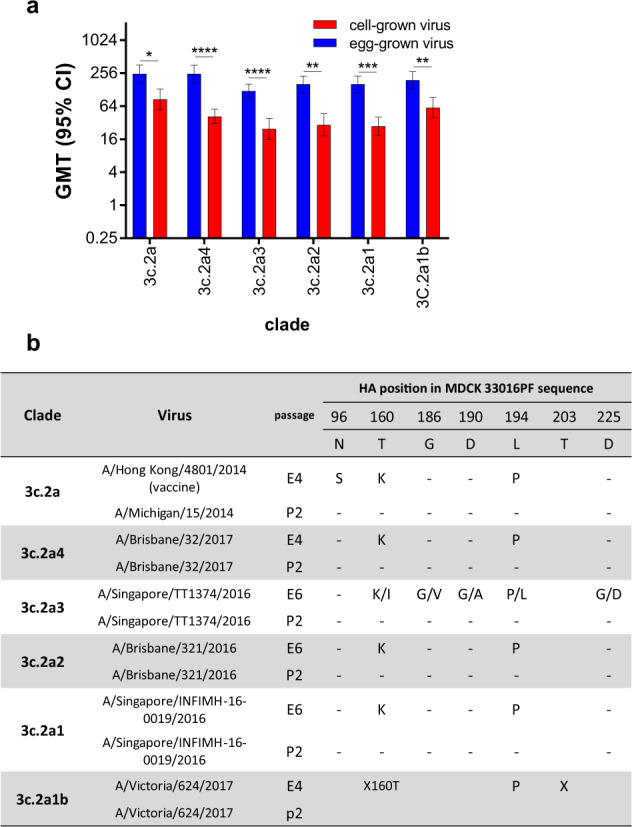
Assessment of impact of egg adaptation on antigenicity. **a** Analysis of post-vaccination serological responses to egg- and cell-grown virus pairs. Twenty human post-vaccination sera were tested by HI assay against the indicated virus. The Geometric Mean Titre (GMT) and 95% confidence interval was calculated for each virus assessed. **b** Amino acid mutations in the HA-1 sequence, as compared to the cell sequence is shown for each virus included in (**a**). *p < 0.05, **p < 0.01, ***p < 0.001, ****p < 0.0001. The difference in GMT was assessed using a multiple *t*-test.

Antibody responses expressed as geometric mean titres to egg-grown viruses were significantly higher than antibody responses to the corresponding cell-grown viruses for all clades tested (Fig. 7a). These data indicate that sera obtained from post-vaccination donors inhibited egg-grown viruses well, but did not inhibit cell-grown viruses well. The egg-grown viruses had all acquired T160K and L194P adaptations in the HA protein, which corresponded to adaptations in the egg vaccine received by the recipients (Fig. 7b). The T160K and L194P mutations are located in antigenic site B of the H3 HA protein. Thus it is likely that the post-vaccine antibody response was directed to the egg adaptations and may not protect as well against infection with circulating viruses, which do not contain these adaptions in the HA protein.

## Discussion

This study demonstrated that human influenza viruses were more readily isolated in the qualified mammalian cell line, MDCK33016PF, than in embryonated hen’s eggs. All subtypes and lineages were isolated at higher levels in MDCK33016PF cells and this remained consistent over the study period (2008–2020), while the isolation rate of viruses in embryonated eggs varied for each subtype and lineage. Changes in receptor binding preferences may influence the isolation of human influenza viruses, especially A(H3N2) viruses, in eggs^24,37^. Viruses that were isolated in the MDCK33016PF cell line had few mutations in the HA, whilst egg adaptation of the HA was common. The egg HA adaptations affected the antigenicity of the virus and reduced the recognition of vaccine-induced antibodies to circulating virus strains as demonstrated in this study and others^38–40^.

The difference in the isolation rate between cells and eggs is significant. Overall, for every five viruses inoculated into cells, on average four will grow, whilst for every five viruses inoculated into eggs, on average only two will grow. Thus at least twice as many inoculations into eggs compared to cells, are required to isolate the same number of viruses. This increased isolation rate in cells is directly related to the susceptibility of each substrate to influenza virus infection with human viruses having to undergo adaption to the avian (egg) host in order to replicate efficiently. Limited isolation of viruses in eggs reduces the choice and availability of viruses for influenza virus reassorting and vaccine manufacture. Poor yielding egg-derived viruses may also lead to a delay in vaccine availability due to the failure to have a high-yielding CVV available. The significantly improved isolation rates in MDCK33016PF cells increase the number of CVVs available for manufacture, and this increases the likelihood that a good growing, high yielding CVV will be available. Furthermore, the reduced number of mutations of cell CVVs compared to egg-grown CVVs indicates that cell-derived isolation results in a better-matched CVV to the circulating influenza viruses, especially with the A(H3N2) subtype.

The improved isolation rate of cells versus eggs may be explained by the level of sialylated N-glycan expression. Human influenza viruses bind to sialylated N-glycans and phosphorylated non-sialylated N-glycans expressed on the human respiratory tract^41–44^. Studies using the Sambucus nigra (SNA) and Maackia amurensis (MAA) lectins show that human respiratory tissues express α2,6-linked sialylated N-glycans more abundantly than α2,3-linked sialylated N-glycans on cells, though both linkage types are present^42,43^. Similar patterns were observed on MDCK cells^45^. Studies of avian tissues show expression of both types of receptors, with an increased level of α-2,3-linked sialylated glycans in tissues^46,47^ and in the chorioallantoic and amniotic membranes in embryonated chicken eggs^48^. An increased expression of human-like surface receptors (α2,6-linked sialylated N-glycans) on MDCK33016PF cells as compared to eggs may explain their improved isolation rates of human influenza viruses. Increased expression of human-like surface receptors may lead to an increase in the avidity of the interaction between the viral HA and its receptor, improving isolation of samples with low amounts of virus or low HA affinity for its receptor^9^. In fact, we showed that influenza A samples isolated in cells only had lower viral loads in the OCS than influenza A samples that could be isolated in both cells and eggs. Over the course of the study, A(H3N2) viruses lost their ability to agglutinate turkey RBC and this directly correlated with a reduction of isolation of viruses in eggs, but not in cells. Others have also documented the loss of turkey RBC agglutination of A(H3N2) viruses over time and the accompanying reduction in HA affinity and specificity for its receptor^24,49–51^. Taken together, these data indicate that MDCK33016PF cells support the isolation of samples by increasing the avidity of the viral HA and the receptor on the cell surface.

This study demonstrated that viruses that are isolated in the MDCK33016PF cell line have fewer mutations in the HA gene, whilst egg adaptation mutations in the HA gene are common. Not unexpectedly, the majority of mutations occurred around the globular head of the HA, the region involved in virus binding to its cell receptor. For cell isolations of influenza viruses, 25% of mutations were located in antigenic sites, and 35% of mutations were located in antigenic sites or the RBS. Mutations were spread over the whole HA, consistent with random mutation due to a lack of proof-reading ability in the polymerase of influenza virus^52^. In contrast, for egg-derived influenza virus isolates, mutations were predominantly in the antigenic sites and RBS (63%). Mutations were particularly focused in these regions for the H3 HA (80% of mutations) and influenza B HA of egg-grown viruses (100% of mutations). It is well known that egg adaptations in A(H3N2) viruses can affect antigenicity as well as receptor binding specificity and affinity^24,36,53^. Changes in affinity or specificity of HA binding can lead to compensatory NA sequence changes^54^. Compensatory NA mutations in egg-passaged viruses predominantly occurred when HA adaptation occurred at the receptor-binding site or surrounding antigenic sites.

Some egg adaptations will lead to detrimental antigenic changes in the HA. Following vaccination with egg-adapted viruses, antibodies may be focussed on the egg-adapted HA sites and not be able to neutralise circulating viruses leading to reduced protection following vaccination with egg-based vaccines. This was demonstrated in this study using a panel of post-vaccination (egg-based vaccine) human sera. The sera recognised egg-grown viruses well, but not cell-grown viruses, which more closely resemble circulating influenza viruses. Similar results with human and animal post-vaccination and infection sera has also been demonstrated by others^19,38^. A significant effort is made to minimise the use of virus strains with known egg adaptations in the manufacture of influenza vaccines but this also restricts the number of egg-based CVVs that are available. However, a certain level of adaptation is tolerated as with many viruses some egg adaptive HA and NA changes are inevitable. The level to which this contributes to reduced vaccine efficacy depends on the virus, the subtype, the lineage, and the year in which the egg CVV and the influenza vaccine was produced. To date, this reduced efficacy has been mostly attributed to influenza A(H3N2) viruses^25–27,55^. In 2017/2018 Northern Hemisphere, Flucelvax^®^ Quadrivalent was available and contained the A(H3N2) component that was exclusively derived and manufactured from a cell CVV. Improved effectiveness of this vaccine component as compared to egg-passaged influenza vaccine in preventing influenza-like illness was demonstrated in the 2017/2018 NH season, where A(H3N2) viruses dominated^22^. All four influenza viruses for Flucelvax^®^ Quadrivalent have been isolated and propagated exclusively in MDCK33016PF cells since 2019/2020, and further improvements in vaccine efficacy are expected. There is also a need to develop alternate technologies, such as cell, recombinant or egg-based platforms that do not allow for egg adaptation^5,29^. The potential for 'humanised-eggs', where eggs express the SIAT1 gene are an alternative future approach. The data in this study clearly demonstrate that the successful isolation of human influenza viruses in MDCK33016PF cells offers an alternate, attractive strategy for CVV generation with the potential for improved influenza vaccine protection.

## Methods

### Clinical specimens and viruses

Human clinical samples that are supplied to the WHO Collaborating Centre for Reference and Research on Influenza in Melbourne (the Centre) fall under the terms of reference of the WHO Global Influenza Surveillance and Response System (GISRS, see ref. ^56^) and the use of these samples for influenza vaccine development is permitted without additional ethics approval being held by the Centre. Human seasonal influenza-positive OCS and virus isolates received from 2008–2020 as part of routine influenza surveillance activities from WHO National Influenza Centres, WHO Collaborating Centres and other regional laboratories and hospitals in Australia, New Zealand and Asia/Pacific region, were included in this study. OCS were predominantly throat swabs, nasal swabs and nasopharyngeal swabs as well as a small number of nasopharyngeal aspirates with very few bronchoalveolar lavages. The clinical samples used in this study were chosen to ensure a broad representation of the viral genetic clades present in each type/subtype circulating at the time.

### Virus infection and passaging in embryonated hen’s eggs

OCS were diluted 1:2(v/v) in a 1000 U/ml neomycin sulfate (Pharmacia & Upjohn)/2000 U/ml polymyxin B sulfate (Xella Pharmaceutical) solution and 200 µl was inoculated into either the amniotic cavity of 13–15 day old embryonated hen’s eggs or the allantoic cavity of 10–12 day old embryonated hen’s eggs (kindly provided by Seqirus). Inoculated eggs were incubated in a humidified incubator at 35 °C for 3–4 days for influenza A viruses and 33 °C for 3–4 days for influenza B viruses. Amniotic or allantoic fluid was harvested and the presence of virus was assessed by Haemagglutination (HA) Assay using 1% turkey or GP RBC. Viruses were passaged up to seven times in eggs, with up to three (blind) passages in the amniotic cavity and up to four passages in the allantoic cavity. For passage, the inoculum was prepared at three different dilutions, into 3–6 eggs per dilution, according to the HA titre of the prior passage (4–16 HA— diluted 10^−1^, 10^−3^, 10^−5^, 32–512 HA—diluted 10^−2^, 10^−4^, 10^−6^, >512 HA—diluted 10^−4^, 10^−6^, 10^−8^ prior to infection).

### Passaging of MDCK33016PF cells

MDCK33016PF suspension cells (a proprietary cell line developed by Novartis^31–33^ and now owned by Seqirus Limited) were maintained at densities between 1–1.5 × 10^6^ cells/ml in 500 ml disposable spinner flasks (Corning, USA) in MDCK 33016 CDM (Lonza, Germany) at 37 °C, 4.5% CO_2_, shaking at 1 g. Cells were passaged at 3–4 day intervals.

### Virus infection in MDCK33016PF cells

Virus infections in cells were performed in a 5 ml total volume in 50 ml filter tubes (TPP, Transadingen, Switzerland), containing 1 × 10^6^ cells/ml (range 9.9–1.1 × 10^6^) in a (7:3 v/v) mixture of PFM:CDM infection media (PFM manufactured by Invitrogen, USA, CDM by Lonza, Germany) supplemented with trypsin (1%, Roche, USA) and neomycin (37.5 µg/ml, Sigma, USA). About 50 µl OCS (neat) was inoculated for passage 1 (P1) inoculation, resulting in a 10^−2^ dilution. If less than 50 µl OCS was available, the specimen tube was washed with 50 µl PBS and this was inoculated into the cells. The virus cell suspension was cultured at 34.5 °C, 5% CO_2_, shaking at 4 g for 72 ± 2 h. To harvest the virus, samples were centrifuged at 500×*g* for 10 min, and the supernatant was collected. The presence of the virus was assessed by HA Assay and flow cytometry (see below). A second passage (P2) of 100 µl P1 virus into 10 ml cells was performed for all samples, as above. The P1 virus inoculum was diluted based on the HA titre (0–8 HA—diluted 1/10; >8–64 HA—diluted 1/100; >64–512 HA—dilution 1/1000; >512 HA—dilution of 1/10000). All samples were stored at −80 °C unless immediately passaged or analysed. All analyses were performed on P2 virus samples.

### Assessment of virus samples by HA Assay and flow cytometry

Egg- and cell-grown virus isolates were assessed for virus growth by HA assay. Briefly, 1% haematocrit turkey or GP RBC was added to an equal volume of virus that had been serially diluted twofold in a microtitre plate^3^.

Virus-infected cells (a total of 10^4^ cells), at 72 h post-inoculation, were fixed and permeabilised using the Cytofix/Cytoperm kit (BD, USA) as per the manufacturer’s instructions. Expression of influenza A and B nucleoprotein (NP) was detected using the IMAGEN influenza A and B kit (Cat # K610511-2, Thermo Scientific, USA), diluted 1:100. Cells were analysed using a FACsCanto II (BD, USA) with FACSDiva™ software (v9, USA). Single cells were identified by forward and side scatter and the proportion influenza-positive was determined by assessment of fluorescence as compared to a ‘mock-infected’ cell population. Isolates were classified as positive for growth in cells or eggs if a HA titre of at least 2 was observed for either turkey or GP RBC. Isolates were also classified as positive for growth in cells if at least 20% of cells were positive for NP by flow cytometry in the absence or presence of a detectable HA titre. There was a high positive correlation between flow cytometry results and HA titres for all subtypes/lineages (see Supplementary Information, Supplementary Fig. 2).

### Sequencing of HA genes

RNA was extracted from 140 µl virus isolate or OCS using QIAmp Viral RNA mini kit (Qiagen, Germany) according to the manufacturer’s instruction. A 5ul aliquot of RNA was used to amplify the HA-1 domain of influenza HA using SuperScript III One-Step RT-PCR with Platinum Taq (Invitrogen, USA) with M13 tagged-gene-specific primers^57^. Unincorporated primers and dNTPs were removed using ExoSAP-IT (Affymetrix, USA), according to the manufacturer’s instructions. DNA sequencing was performed with M13 primers (M13F-59 TGTAAAACGACGGCCAGT and M13R 59 CAGGAAACAGCTATGACC) in a 96-well plate format using the BigDye Terminator v3.1 Cycle Sequencing Kit (Thermo Scientific, USA), followed by the removal of excess dye terminators with a BigDye XTerminator purification kit (Thermo Scientific, USA). The sequence was determined using an automated capillary DNA sequencer (ABI Prism 3500xL). Identification of mixed bases was set at 25%. Sequences were assembled as consensus using DNASTAR Lasergene Suite v.9.1.0, Seqman v9.1 and compared using Megalign (v9.1).

### Haemagglutination inhibition assay

Pre- and post-vaccination sera were used to analyse antibody responses. Subjects were enroled in a clinical trial at the Royal Adelaide Women and Children’s hospital, and blood was drawn prior to influenza vaccination and 21 days post-vaccination. Subjects were vaccinated with the Seqirus Southern Hemisphere quadrivalent vaccine for the 2016 season (the vaccine contained an A/California/7/2009-like virus, an A/Hong Kong/4801/2014-like virus and a B/Brisbane/60/2008-like virus). Sera was sent to the WHO CC in Melbourne, and antibody responses to the four vaccine components were assessed using the Haemagglutination inhibition (HI) assay using both egg and cell-derived viruses^3^. Serum samples were pretreated with receptor destroying enzyme II (Denka Seiken Co. Ltd.), 1:5 (volume/volume). Influenza virus (4 HA units, 25 μl) was added to an equal volume of treated serum that had been serially diluted twofold in a microtitre plate. Following a 1-h incubation, 25 μl 1% (volume/volume) guinea pig RBC was added to each well. HI was read after 45 min. Titres were expressed as the reciprocal of the highest dilution of serum where Haemagglutination was inhibited.

### Statistical analysis

The proportion of viruses isolated in eggs as compared to cells was assessed using Chi-squared analysis. The difference in variation in isolation rates over the years studied between egg and cells was assessed using the *F*-test, calculated in Excel (Microsoft, Australia). The difference in HA titres or Cts between viruses isolated only in cells as compared to viruses isolated in both cells and eggs was assessed using an unpaired *t*-test, using GraphPad Prism Version 7. The difference in HI titres between cell and egg-grown isolates was assessed using a multiple *t*-test, using GraphPad Prism Version 7.

### Reporting Summary

Further information on research design is available in the Nature Research Reporting Summary linked to this article.

## Supplementary information

Supplementary Information

Reporting Summary

## Data Availability

The datasets generated during and/or analysed during the current study are available from the corresponding author on reasonable request.
