# Supplementary material for: Enhanced isolation of influenza viruses in qualified cells improves the probability of well-matched vaccines
Source: NPJ Vaccines. 2021 Dec 9;6:149. doi: 10.1038/s41541-021-00415-3 (PMC8660794; doi:10.1038/s41541-021-00415-3)
Supplement: Supplementary file 1 — Supplementary Information [file 41541_2021_415_MOESM1_ESM.pdf]

Supplementary Information

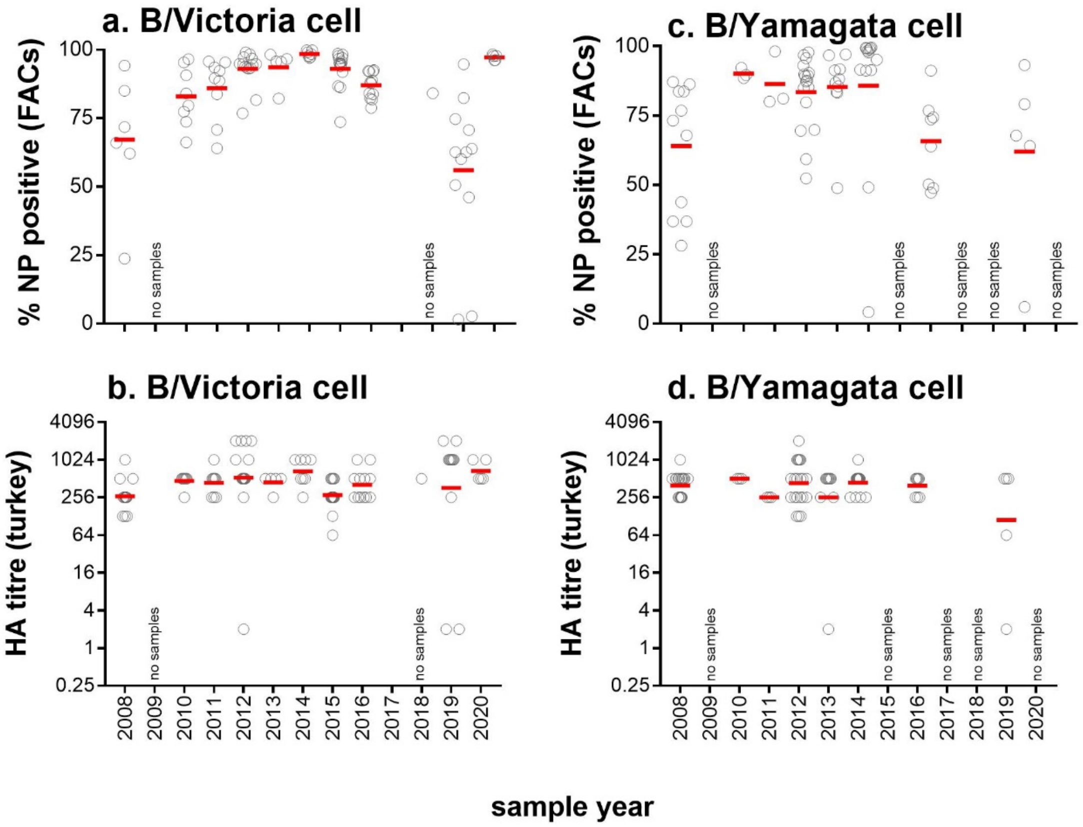

Supplementary Figure 1. HA titres of influenza B isolates from 2008-2020. (**a,c**) Cell-grown B/Victoria and B/Yamagata viruses were assessed for infectivity of cells by flow cytometry. Red bar indicates mean. (**b,d**) HA titres of cell-grown isolates to turkey RBC. Red bar indicates geometric mean titre.

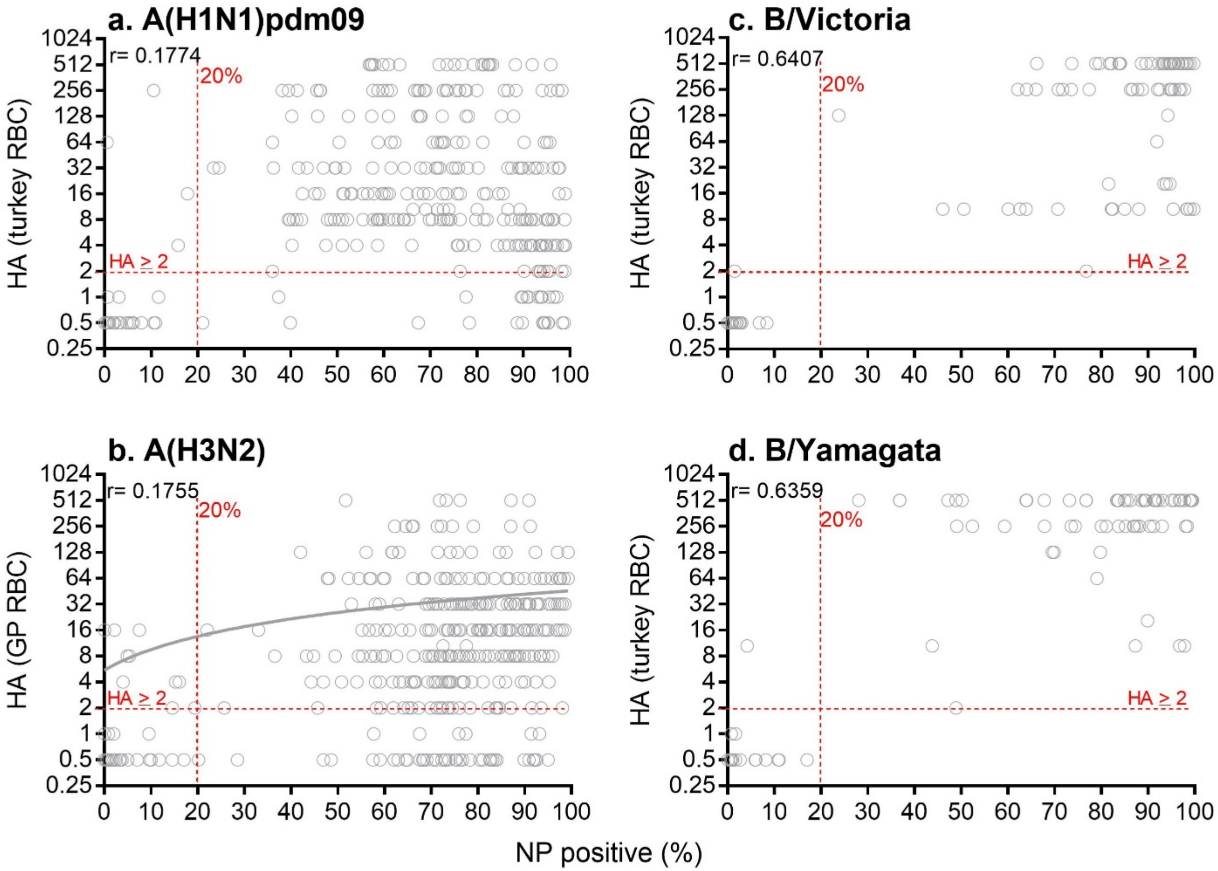

Supplementary Figure 2. The correlation between proportion NP-positive (%) by flow cytometry and HA titre was plotted for cell-grown virus isolates, by subtype/lineage. The horizontal dotted line indicates a HA of at least 2 and the vertical dotted line indicates 20% positivity by flow cytometry.

Supplementary Table 1. Isolation rates in cells and eggs by year and subtype/lineage.

year	A(H1N1)						A(H3N2)						B/Victoria						B/Yamagata					
	cells			eggs			cells			eggs			cells			eggs			cells			eggs		
	# positive	# attempted	%	# positive	# attempted	%	# positive	# attempted	%	# positive	# attempted	%	# positive	# attempted	%	# positive	# attempted	%	# positive	# attempted	%	# positive	# attempted	%
2008	0	0	n/a	0	0	n/a	7	7	100	6	7	86	15	22	68	3	15	20	22	29	76	2	4	50
2009	20	23	87	17	23	74	19	21	90	14	18	78	0	0	n/a	0	0	n/a	0	0	n/a	0	0	n/a
2010	23	35	66	11	19	58	21	23	91	8	16	50	8	8	100	7	8	88	3	5	60	0	1	0
2011	16	33	48	9	26	35	28	38	74	4	31	13	9	12	75	1	6	17	3	3	100	2	5	40
2012	18	19	95	4	19	21	35	38	92	10	38	26	17	21	81	6	19	32	21	26	81	3	23	13
2013	43	54	80	17	51	33	36	47	77	19	47	40	5	6	83	1	6	17	10	14	71	5	14	36
2014	14	20	70	10	20	50	62	83	75	23	83	28	8	11	73	2	11	18	14	16	88	5	16	31
2015	41	49	84	18	49	37	39	42	93	19	42	45	16	21	76	6	21	29	0	0	n/a	0	0	n/a
2016	36	43	84	12	23	52	61	68	90	15	61	25	12	16	75	4	5	80	8	9	89	4	5	80
2017	40	48	83	6	8	75	49	56	88	21	56	38	0	0	n/a	0	0	n/a	0	0	n/a	0	0	n/a
2018	41	44	93	17	17	100	44	50	88	17	22	77	1	5	20	3	5	60	0	0	n/a	0	0	n/a
2019	31	48	65	4	4	100	45	72	63	13	27	48	12	20	60	5	6	83	5	5	100	3	4	75
2020	11	18	61	2	2	100	16	32	50	4	11	36	5	5	100	1	1	100	0	0	n/a	0	0	n/a

Supplementary Table 2. Amino acid mutations in HA and NA proteins of viruses following passage in eggs or cells. Only viruses with mutation in the HA protein are listed.

Changes in HA and NA proteins (aa) after cell and egg passage							
**Subtype/lineage**	**Virus**	**MDCK passage**			**egg passage**		
		**HA[a]**	**NA[a]**	**passage**	**HA[a]**	**NA[a]**	**passage**
A(H1N1)	A/NEWCASTLE/7/2013	-	-[b]	P2	Q223R	-	E4
A(H1N1)	A/CHRISTCHURCH/6/2014	-	-[b]	P2	S164X, S183P, Q223X	-	E3
A(H1N1)	A/FIJI/2/2016	-	N270K	P2	Q223R	N270K	E3
A(H1N1)	A/FIJI/3/2016	-	-	P2	Q223R	-	E3
A(H1N1)	A/FIJI/8/2016	-	-	P2	L191I	-	E3
A(H1N1)	A/VICTORIA/900/2016	-	-[b]	P2	L191X, Q223X	-	E3
A(H1N1)	A/DARWIN/20/2015	-	-[b]	P2	L191X, D222X	S37X[b]	E3
A(H1N1)	A/FIJI/17/2015	-	-[b]	P2	K43X, L191I	-	E3
A(H1N1)	A/BRISBANE/10/2010	-	-[b]	P2 (P3[e])	D127E	-	E2
A(H1N1)	A/DARWIN/2126/2009	-	-[b]	P2 (P3[e])	S183P	D248X[b]	E2
A(H1N1)	A/VICTORIA/528/2009	-	-[b]	P2 (P3[e])	V30G, L191I	S12L	E3
A(H1N1)	A/VICTORIA/2083/2009	-	-[b]	P2 (P3[e])	L191I	S12L, D248N[b]	E2
A(H1N1)	A/DARWIN/2131/2009	-	-	P2	E21K, G225A	-	E2
A(H1N1)	A/GUAM/2050/2009	-	-[b]	P2	N10Y, Q223R	-	E2
A(H1N1)	A/VICTORIA/523/2012	-	-[b]	P2	R45E, D127E, S183X, G225X	I188X[b]	E5
A(H1N1)	A/DARWIN/70/2011	T82X	n/a[c]	P2	L191X	n/a[c]	E3
A(H1N1)	A/WELLINGTON/1/2011	-	n/a[c]	P2	Q223R	n/a[c]	E3
A(H1N1)	A/BRISBANE/190/2011	-	n/a[c]	P2	Q223R	n/a[c]	E3
A(H1N1)	A/MALAYSIA/478/2011	X131G, X155E, X183P	n/a[c]	P2	X131G, X155E, X183P	n/a[c]	E2

A(H1N1)	A/MALAYSIA/517/2011	X385R, X509M	n/a[c]	P2	D222G, X385K, X509M	n/a[c]	E3
A(H1N1)	A/PERTH/533/2011	-	n/a[c]	P2	Q223R	n/a[c]	E3
A(H1N1)	A/WELLINGTON/1/2010	-	n/a[c]	P2	S183P	n/a[c]	E2
A(H1N1)	A/WELLINGTON/4/2010	-	n/a[c]	P2	L191I	n/a[c]	E2
A(H1N1)	A/GUAM/2/2010	-	n/a[c]	P2	S183P	n/a[c]	E2
A(H1N1)	A/TOWNSVILLE/121/2011	T197X	n/a[c]	P2	not done[d]	n/a[c]	
A(H1N1)	A/VICTORIA/528/2012	L191X	n/a[c]	P2	not done[d]	n/a[c]	
A(H1N1)	A/VICTORIA/24/2012	S183X	n/a[c]	P2	not done[d]	n/a[c]	
A(H1N1)	A/WELLINGTON/2/2012	L191X	n/a[c]	P2	not done[d]	n/a[c]	
A(H3N2)	A/PORT MORESBY/2/2009	-	n/a[c]	P2	Y161S, G186V, N498S	n/a[c-]	E3
A(H3N2)	A/TOWNSVILLE/87/2010	-	-[b]	P2	I140M, L194P	-[b]	E3
A(H3N2)	A/CHRISTCHURCH/28/2011	-	-[b]	P2	G186V, S219Y	-[b]	E5
A(H3N2)	A/VICTORIA/362/2011	-	-[b]	P2	H183X	-[b]	E3
A(H3N2)	A/CHRISTCHURCH/512/2012	-	-[b]	P2	G186V, N246H	V50M[b]	E6
A(H3N2)	A/BRISBANE/198/2013	-	-[b]	P2	L194P	-[b]	E4
A(H3N2)	A/PERTH/239/2013	-	-[b]	P2	L194P, T203I	-[b]	E4
A(H3N2)	A/PERTH/98/2013	-	-[b]	P2	G186V, S219X	-[b]	E4
A(H3N2)	A/VICTORIA/710/2013	-	-[b]	P2	S145N, L194P	-[b]	E5
A(H3N2)	A/BRISBANE/341/2014	-	-[b]	P2	T160K, L194P, T203I	-[b]	E4
A(H3N2)	A/BRISBANE/71/2015	X338F, X366D	-[b]	P2	T160K, L194P, X338F, X366D	-[b]	E5
A(H3N2)	A/FIJI/2/2015	-	-[b]	P2	T160K, L194P, D225G	-[b]	E5
A(H3N2)	A/FIJI/4/2015	-	-[b]	P2	T160K, L194P, D225G, S262N	-[b]	E5
A(H3N2)	A/FIJI/7/2015	-	-[b]	P2	T160K, L194P	C53F[b]	E5
A(H3N2)	A/NEW CALEDONIA/71/2014	-	-[b]	P2	T160K, L194P, D225G	-[b]	E5

A(H3N2)	A/SOUTH AUSTRALIA/21/2015	-	-[b]	P2	T160K, L194P, T203X	-[b]	E4
A(H3N2)	A/SOUTH AUSTRALIA/9/2015	-	-[b]	P2	I140X, T160K, L194P	-[b]	E5
A(H3N2)	A/VICTORIA/5006/2014	T160X	-[b]	P2	Y94X, T160K, L194P	-[b]	E4
A(H3N2)	A/VICTORIA/6001/2015	-	-[b]	P2	G186V, S219X, T248X, A372S	-[b]	E4
A(H3N2)	A/VICTORIA/673/2014	-	-[b]	P2	N96S, T160K, L194P	-[b]	E4
A(H3N2)	A/VICTORIA/505/2013	-	-[b]	P2	G186V, S219F	-[b]	E4
A(H3N2)	A/SOUTH AUSTRALIA/24/2014	-	-[b]	P2	H156R, D225G	-[b]	E4
A(H3N2)	A/SOUTH AUSTRALIA/40/2014	-	-[b]	P2	G186V, N246H	-[b]	E3
A(H3N2)	A/SOUTH AUSTRALIA/55/2014	E62X	-[b]	P2	S145N, D225G	-[b]	E5
A(H3N2)	A/NEWCASTLE/25/2014	-	-[b]	P2	G186V, S219X, N246X	-[b]	E3
A(H3N2)	A/VICTORIA/511/2015	-	-[b]	P2	I140K, H156Q, G186X, S219F	-[b]	E3
A(H3N2)	A/TOWNSVILLE/101/2010	P11A	n/a[c]	P2	not done[d]	n/a[c]	
A(H3N2)	A/NEWCASTLE/55/2010	C473R	n/a[c]	P2	not done[d]	n/a[c]	
A(H3N2)	A/SINGAPORE/13/2011	A212S, N225D, S497N	n/a[c]	P2	not done[d]	n/a[c]	
A(H3N2)	A/PERTH/39/2015	T160K	n/a[c]	P2	not done[d]	n/a[c]	
A(H3N2)	A/SOUTH AUSTRALIA/46/2014	E62X	n/a[c]	P2	not done[d]	n/a[c]	
A(H3N2)	A/SOUTH AUSTRALIA/49/2015	R33Q, E62K, T160K	n/a[c]	P2	not done[d]	n/a[c]	
A(H3N2)	A/AUCKLAND/20/2013	N225D	n/a[c]	P2	not done[d]	n/a[c]	
B/Yamagata	B/HONG KONG/2205/2010	-	n/a[c]	P2	N196X	n/a[c]	E3
B/Yamagata	B/VICTORIA/500/2011	-	n/a[c]	P2	N196D	n/a[c]	E2

B/Yamagata	B/SOUTH AUSTRALIA/76/2013	-	-[b]	P2	N196X	-[b]	E4
B/Yamagata	B/DARWIN/20/2014	-	-[b]	P2	N196D	-[b]	E3
B/Yamagata	B/DARWIN/13/2014	-	-[b]	P2	N196Y	-[b]	E3
B/Victoria	B/VICTORIA/512/2010	-	n/a[c]	P2	N197S	n/a[c]	E2
B/Victoria	B/SYDNEY/508/2010	-	n/a[c]	P2	N197S	n/a[c]	E2
B/Victoria	B/BRISBANE/18/2013	-	-[b]	P2	N197X	-[b]	E4
B/Victoria	B/VICTORIA/18/2012	-	-[b]	P2	N197S	-[b]	E4
B/Victoria	B/SOUTH AUSTRALIA/36/2012	-	-[b]	P2	T199S	-[b]	E3
B/Victoria	B/SOUTH AUSTRALIA/81/2012	-	-	P2	T199A	-	E4
B/Victoria	B/VICTORIA/849/2015	-	-[b]	P2	T199A	-[b]	E4
B/Victoria	B/NEW CALEDONIA/6/2014	E48X, D64X	-[b]	P2	T199I	-[b]	E3

[a] changes as compared to OCS

[b] changes as compared to cell passage P2

[c] comparison not available as cell-grown and/or egg-grown virus NA protein sequence not available

[d] egg passage not performed

[e] P3 passage sequenced for NA protein
